# Nationwide Survey about the Occurrence of Aspergillosis in Captive Penguins in Zoos and Aquariums in Japan

**DOI:** 10.3390/ani13121913

**Published:** 2023-06-08

**Authors:** Megumi Itoh, Naoya Matsumoto, Kyogo Hagino, Nanako Sawayama, Miki Kuwayama, Kazutaka Yamada, Takahito Toyotome

**Affiliations:** 1 Department of Veterinary Medicine, Obihiro University of Agriculture and Veterinary Medicine, Obihiro 080-8555, Hokkaido, Japan; 2 Noboribetsu Marine Park Nixe, Noboribetsu 059-0492, Hokkaido, Japan; 3 School of Veterinary Medicine, Azabu University, Sagamihara 252-5201, Kanagawa, Japan; 4 Diagnostic Center for Animal Health and Food Safety, Obihiro University of Agriculture and Veterinary Medicine, Obihiro 080-8555, Hokkaido, Japan; 5 Medical Mycology Research Center, Chiba University, Chiba 260-8673, Chiba, Japan

**Keywords:** aspergillosis, Japan Association of Zoos and Aquariums, penguin, soil, survey

## Abstract

**Simple Summary:**

Aspergillosis is a fungal respiratory infectious disease in humans and animals and a major cause of mortality in captive penguins. The causative agents of this disease are members of *Aspergillus* species. These fungi are commonly found in the environment. Therefore, rearing conditions and countermeasures in facilities are important to preventing aspergillosis. However, their efficacy has not been investigated sufficiently. In addition, a nationwide survey on the occurrence of aspergillosis, rearing conditions, and countermeasures has not been performed for several decades in Japan. Therefore, this study evaluated the incidence of and preventive measures against aspergillosis in Japanese zoos and aquariums that rear penguins. The incidence of aspergillosis was significantly high in facilities where penguins were housed outdoors, in contact with soil, or moved outside of the rearing enclosure. Overall, 76% of dead penguins had been at individual risk and 54% had been kept in uncomfortable environments. Aspergillosis was thought to occur when individual risk factors and uncomfortable environmental factors were present along with the risk of *Aspergillus* exposure. Recognizing these risks and implementing appropriate measures to prevent contact with *Aspergillus*-contaminated soil are necessary to further reduce aspergillosis-related mortality in penguins.

**Abstract:**

We surveyed the facilities that were members of the Japan Association of Zoos and Aquariums to clarify the incidence of aspergillosis, which is a major cause of death in captive penguins, and to discern effective preventive measures. Responses were obtained for 2910 penguins in 64 facilities; 73 penguins (2.5%) in 35 facilities had died from aspergillosis during the past 5 years from April 2016 to March 2021. Answers to questions about the rearing environment indicated that aspergillosis occurred significantly more often in facilities where penguins were reared outdoors, were in contact with soil, or were moved outside of the rearing enclosure. Answers to questions about their dead penguins indicated that 76% may have been at individual risk (e.g., young age, old age, molting period, and breeding season) and 54% were thought to be reared in uncomfortable environments (e.g., high temperature, high humidity). Aspergillosis may occur when individual risk factors and uncomfortable environmental factors are added to the risk factors of exposure to *Aspergillus*, such as the presence of soil. These conditions must be recognized as risk factors for aspergillosis, and appropriate preventive measures, such as avoiding penguin contact with the soil where *Aspergillus* is expected to be present, can minimize aspergillosis-related deaths.

## 1. Introduction

There are currently 18 species of penguins prevailing, and all of them are found widely in the Southern Hemisphere from the equator to the Antarctic region. Japan has the largest number of captive penguins, with over 3000 of them [1,2]. Among them, 11 penguin species are reared in zoos and aquariums in Japan. Penguins are reared in 89 of 141 zoos and aquariums belonging to the Japan Association of Zoos and Aquariums (JAZA). The species are roughly classified into Antarctic/sub-Antarctic penguins (emperor penguin (*Aptenodytes forsteri*), king penguin (*Aptenodytes patagonicus*), Adélie penguin (*Pygoscelis adeliae*), gentoo penguin (*Pygoscelis papua*), chinstrap penguin (*Pygoscelis antarctica*), northern rockhopper penguin (*Eudyptes moseleyi*) and southern rockhopper penguin (*Eudyptes chrysocome*)) as well as temperate penguins (little penguin (*Eudyptula minor*), African penguin (*Spheniscus demersus*), Magellanic penguin (*Spheniscus magellanicus*), and Humboldt penguin (*Spheniscus humboldti*)) [3]. As described in the *Penguin Care Manual* by the Association of Zoos and Aquariums (AZA) [3], the optimal air temperature ranges vary according to the penguin species. For Antarctic/sub-Antarctic penguin species, the optimal air temperature ranges from −6 °C to 7 °C. However, the optimal air temperature of temperate penguins ranges from 4.5 °C to 26.5 °C. Therefore, zoos and aquariums should adjust their rearing conditions according to the type of penguin species. In Japan, nationally standardized guidelines for the care of penguins have not been established. Therefore, in addition to the information in the AZA manual, each zoo and aquarium develop care protocols via trial and error.

The *Aspergillus* species [4,5,6,7,8,9,10,11,12,13] are common fungi found in the environment, such as soil and air [14,15,16,17,18,19]. Their spores are hydrophobic and extremely small, with a diameter of approximately 2.5 µm. Therefore, they easily pass through the trachea to reach the air sacs and lungs, which causes aspergillosis, a respiratory infection in immunocompromised humans and animals [4,20]. The three most common species of the *Aspergillus* genus that cause aspergillosis are *A. fumigatus*, *A. niger*, and *A. flavus,* which are also major producers of mycotoxin aflatoxin [21]. In particular, *A. fumigatus* is the most pathogenic species that causes severe respiratory symptoms in wild birds and poultry [21,22]. A large number of aspergillosis cases in free-living birds have been reported in the scientific literature. Converse [21] evaluated 88 published reports, including a case of little penguins in Australia. Hllgarth and Kear [23] revealed that waterbirds in captivity are more susceptible to aspergillosis than land birds. Furthermore, some shore birds are susceptible to aspergillosis.

Aspergillosis is one of the leading causes of death in captive penguins. The major clinical signs are nonspecific, such as inappetence, lethargy, weight loss, coughing, and open-mouth breathing; therefore, distinguishing aspergillosis from other diseases and confirming the diagnosis remains difficult [4,7]. Long-term antifungal therapy is considered effective; however, by the time clinical signs are recognized, the infection has already progressed to the point that it cannot be cured [4,24].

Recently, a combination of serological markers and computed tomography (CT) has proven useful for early diagnosis and therapeutic intervention in aspergillosis [25,26,27]. In a case of aspergillosis in king penguins, aspergillosis was diagnosed by the increased titer of *Aspergillus* antibody in the serum screening test, subsequent CT examination and in turn cured using itraconazole treatment [25]. However, the development of preventive measures against aspergillosis is as crucial as early diagnosis and treatment of aspergillosis in captive penguins.

Aspergillosis is considered an opportunistic infectious disease [4,20]. Therefore, to prevent aspergillosis, contact with *Aspergillus* must be minimized in penguins, and the health of each penguin should be maintained [28,29,30,31].

*Aspergillus* is ubiquitous in soil. Hence, in previous reports, we reported and implemented measures to minimize penguin contact with the soil. For example, the penguins did not take a route with exposed soil during daily penguin marches, and the feet of penguins were washed with seawater after marches. Moreover, the rearing facility was cleaned with hot water and 0.02% hypochlorite. As a result, although the aquarium had six aspergillosis cases between 2009 and 2015, there was no incidence of aspergillosis from 2016 to 2020 [28,29].

Aspergillosis might occur in stressed conditions. Putative stressors have been associated with inadequate heat or cold environment, stressful relocation, breeding, and molting [4]. To the best of our knowledge, surveys of aspergillosis in captive penguins have not been reported in Japan for several decades. However, a survey on captive penguins was performed in 1972 by Inokashira Park Zoo [32]. Therefore, the current situation of aspergillosis among captive penguins in Japan is not clear. In this study, with the cooperation of JAZA and its members, we surveyed the occurrence of aspergillosis among captive penguins in Japan, the rearing environment, the preventive measures implemented, and the risks envisioned by keepers. In addition to reporting the results of the survey, based on them, we propose feasible and effective preventive measures for aspergillosis in captive penguins in Japan.

## 2. Materials and Methods

### 2.1. Primary Survey 

In September 2021, through JAZA, we sent a questionnaire (Supplementary File S1) about aspergillosis in penguins to 89 zoos and aquariums (members of JAZA) that bred penguins. Sixty-four facilities responded to the questionnaire with information on 2910 penguins belonging to 11 species (Table 1), and these data were used in the analysis.

The questionnaire (Supplementary File S1) had the following questions for respondents: (1) species and the number of penguins in each facility; (2) species and the number of penguins that had died of aspergillosis in the 5 years from April 2016 to March 2021; (3) the environment of indoor and outdoor rearing enclosures (indoor or outdoor, temperature, humidity, ventilation, floor material, and nesting material) for each penguin species; (4) whether penguins were moved outside of the rearing enclosure (if so, the purpose, season, frequency, and flooring material of the moving route); (5) measures to prevent aspergillosis; and (6) free-text field for additional data. 

Based on these responses, we initially determined the number and mortality rate of aspergillosis in each penguin species. Thereafter, the number of facilities rearing each penguin species was examined. For penguin species with high mortality rates, the mortality rate in each facility was confirmed. Next, to distinguish the penguins at each facility, each penguin species reared at each facility was counted as a single group. If there were three species of penguins in one facility, the facility was counted as three groups. Each group had exactly the same rearing environment. The association between aspergillosis and the use of outdoor enclosures, movement outside of the enclosures, and the presence of soil in indoor or outdoor enclosures or walking routes was investigated. Analyses were conducted for all the penguin species and Antarctic/sub-Antarctic or temperate penguin species. Additionally, we assessed the measures of aspergillosis implemented at each facility and the association between the number of measures and aspergillosis. Based on the respondent comments written in the free-text field, the risk factors of aspergillosis were determined according to the keepers and veterinarians.

### 2.2. Additional Survey

In the primary survey, breeders and veterinarians predicted the following causes of aspergillosis on this questionnaire: (1) individual conditions, such as association between aspergillosis and transfer from other facilities, old age, young age, molting period, and breeding period; (2) rearing in an uncomfortable environment, such as one with high temperature, high humidity, and poor ventilation; and (3) use of nest boxes and nest materials that are susceptible to mold. Based on these comments, an additional questionnaire (Supplementary File S2) was sent to each facility that reported penguin deaths of aspergillosis in the primary survey. Questions were regarding the month of occurrence of aspergillosis, the sex and age of dead penguins, whether they had been transferred from other zoos or aquariums within the past year, whether the disease occurred during the molting or breeding period, cause of death suspected by respondents (presence of underlying diseases, the possibility of rearing in an uncomfortable environment, etc.), rearing under hot and humid conditions, contact with soil or plants or both, the type of nesting box, and bedding materials. 

Transfer from another facility within the past year, younger than 1 year, older than 20 years, breeding season, molting period, and treatment for a disease other than aspergillosis were aggregated as individual risk factors. Respondents suspected poor hygiene and inadequate ventilation as the causes of death, so high temperature and humidity, poor sanitation, and poor ventilation were aggregated as uncomfortable environmental factors. Moreover, the probability of contact between penguins and soil as a risk factor for exposure to *Aspergillus* spores was aggregated.

### 2.3. Statistical Analysis

Differences in the mortality rate of aspergillosis among penguin species were analyzed using the chi-square test and residual analysis. The associations between aspergillosis and the use of outdoor enclosures, movement outside of the rearing enclosures, and the presence of soil in indoor or outdoor enclosures or walking routes were analyzed using the chi-square test or Fisher’s exact test. According to Cochran’s rule, Fisher’s exact test was used instead of the chi-square test when at least one cell of the 2 × 2 contingency table had <5 as the expected frequency. The association between aspergillosis and the number of preventive measures taken was analyzed using the Wilcoxon rank-sum test. A *p*-value of <0.05 was considered significant. For the statistical analyses, Excel add-in Statcel4 (OMS Publishing Inc., Saitama, Japan) was used.

To evaluate the association between the occurrence of aspergillosis and outdoor enclosures or moving outside of the rearing enclosures, we not only included all penguin species in the analysis but also classified them into two types based on their habitat: Antarctic/sub-Antarctic (emperor penguin, king penguin, Adélie penguin, gentoo penguin, chinstrap penguin, and northern and southern rockhopper penguins) and temperate (little penguin, African penguin, Magellanic penguin, and Humboldt penguin) [3]. According to the *Penguin Care Manual* by AZA, Antarctic and sub-Antarctic penguin species need to be reared in climate-controlled indoor rooms. Temperate species can be reared indoors or outdoors, or both [3].

## 3. Results

### 3.1. Incidence of Aspergillosis among Captive Penguins

We received responses from 64 facilities (72.0%). These facilities were in various regions of Japan (Hokkaido, Honshu, Shikoku, and Kyushu, but not in Okinawa prefecture). Each facility had from 1 to 9 species of penguin, and all the facilities had a total of 2910 penguins. Eleven penguin species were in these facilities (Table 1)

Of the 64 facilities that responded to the questionnaire, 35 facilities reported penguin deaths from aspergillosis in the 5 years from April 2016 to March 2021; of the 2910 penguins, 73 (2.5%) died. No emperor penguins, chinstrap penguins, or northern rockhopper penguins died, and only one Adélie penguin died. Statistically higher death rates were reported among southern rockhopper (11.4%), little (10.2%), and Magellanic penguins (33.3%; Table 2). The death of five southern rockhopper penguins and four little penguins occurred in only one of seven facilities and one of four facilities, respectively, and these deaths occurred in one facility rearing both species. Furthermore, although 13 deaths of Magellanic penguins were reported in 7 facilities of the survey, 4 of 13 deaths occurred at a single facility. By analysis, excluding these facilities, there is no significant difference in death rate by species. 

### 3.2. Relationship between Rearing Environment and Incidence of Aspergillosis

Next, we focused on the rearing environments of each penguin species at each facility. Based on the results of the survey regarding the rearing environments, the groups that used outdoor enclosures (35 (47%) of 74) had a significantly higher incidence of aspergillosis than the groups that used indoor enclosures (6 (13%) of 45 groups; *p* < 0.01). Furthermore, the groups that moved outside of the rearing enclosures (19 (54%) of 35) had a significantly higher incidence of aspergillosis than the groups that did not move outdoors (22 (26%) of 84; *p* < 0.01). Penguins that come in contact with soil had a significantly high incidence of aspergillosis (20 (63%) of 32; *p* < 0.01) (Table 3). The main reason for moving penguins outside of the rearing enclosures was for walking exercise (68% of groups).

To validate whether the significances of environmental factors differed between Antarctic/sub-Antarctic penguin species and temperate penguin species, we roughly classified penguin species into two types and analyzed them. Among the Antarctic/sub-Antarctic penguin species, emperor, Adélie, chinstrap, and northern rockhopper penguins were housed indoors only. King, gentoo, and southern rockhopper penguins were reared outdoors only during winter in temperate regions or year-round in Hokkaido (the coolest island in Japan). Of the temperate species, those reared outdoors were little penguins (50% of groups), African penguins (78% of groups), Magellanic penguins (92% of groups), and Humboldt penguins (95% of groups).

Most Antarctic/sub-Antarctic penguins were reared indoors, and the instructions provided in the *Penguin Care Manual* by AZA were followed (Table 4). Aspergillosis occurrence in Antarctic/sub-Antarctic penguins reared in outdoor enclosures was significantly high compared to those in indoor enclosures (*p* = 0.007; Table 4). Penguins that had contact with soil had a higher incidence of aspergillosis (2 of 3 groups) than those that did not have contact with soil in the rearing environment (6 of 43 groups). However, there was no significant difference between with and without aspergillosis groups (*p* = 0.073; Table 4).

Most temperate species were reared in outdoor enclosures (Table 5). The occurrence of aspergillosis was significantly high in groups of penguins with increased chances of moving outside of the rearing enclosures (*p* = 0.009) and in groups of penguins having contact with the soil (*p* = 0.019).

### 3.3. Relationship between Incidence of Aspergillosis and Preventive Measures

**Figure 1:**
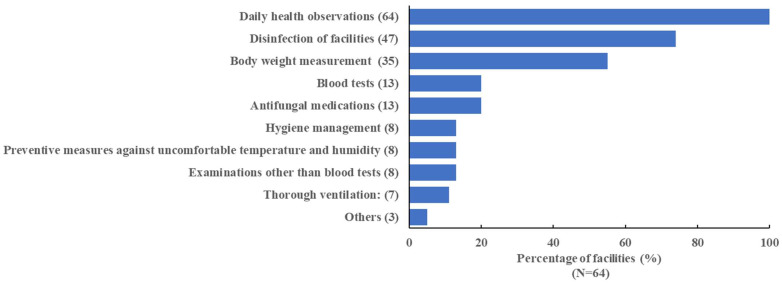
Measures implemented to prevent aspergillosis. Disinfection of facilities: floor, wall, or pool of rearing enclosures. Blood tests: *Aspergillus* antigen or antibody, white blood cell count, protein fraction. Hygiene management: foot-wear disinfection, change in boots, airspace disinfection. Preventive measures against uncomfortable temperature and humidity: water spraying, dehumidifier, and blower. Examinations other than blood tests: culture of environmental microbes or throat swabs, radiography, and CT scan examination. Others: discontinuing the use of groundwater and replacing the soil. The number of facilities taking each measure is shown in parentheses. There are 64 facilities included in the figure.

**Figure 2:**
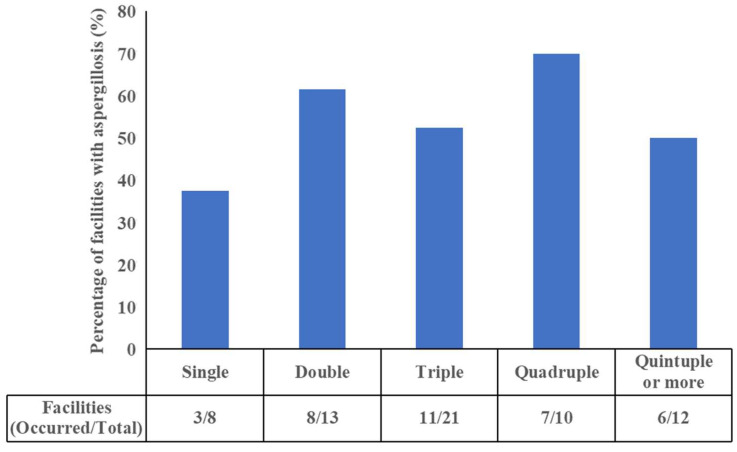
The number of measures implemented to prevent aspergillosis and the incidence of aspergillosis. The values shown below the bar graph are the number of facilities where aspergillosis occurred and the number of facilities included in each category.

We examined the measure to prevent aspergillosis in rearing facilities (Figure 1). Daily health observations were performed at every facility. Furthermore, the following measures were performed as preventive measures against *Aspergillus* infection: disinfection of floors, pools, and walls in each rearing enclosure (74% of the facilities), body weight measurement (55% of the facilities), blood tests (20% of the facilities), antifungal medications for prophylaxis or in individual penguins suspected of having aspergillosis (20% of the facilities), hygiene management (such as foot-wear disinfection, change of boots, and airspace disinfection; 13% of the facilities), prevention measures against uncomfortable temperature and humidity (13% of the facilities), examinations other than blood tests (culturing environmental microbes or throat swabs, radiography; 13% of the facilities), and thorough ventilation (11% of the facilities). Discontinuing the use of groundwater and replacing the soil were reported as other measures according to the answers provided by respondents. Twelve respondents described taking five or more measures to prevent aspergillosis. We examined the association between the number of measures and incidence of aspergillosis. Results showed that aspergillosis occurred in approximately 50% of the facilities. No significant reduction in the incidence of aspergillosis was detected in facilities with five or more measures (Figure 2). 

### 3.4. Verification of Risk Factors and the Environment for the Occurrence of Aspergillosis in Captive Penguins

**Figure 3:**
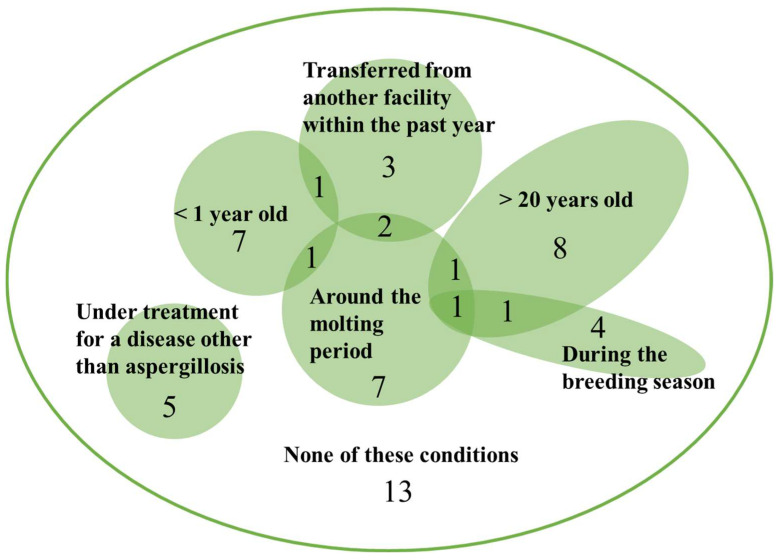
Putative individual risk factors in 54 penguins dead from aspergillosis.

Of all the facilities, 35 zoos and aquariums reported penguin deaths from aspergillosis in the primary survey. A total of 30 of 35 facilities responded to the additional survey regarding the penguins that died from aspergillosis (Supplementary File S3). Keepers and veterinarians responded regarding the putative risk factors in penguins dying from aspergillosis in the survey. Of the 54 penguins, 41 (76.0%) had one or more of the following putative individual risk factors: penguins that died being transferred from another facility within the past year (6 penguins), older than 20 years (11 penguins), younger than one year (9 penguins); having died during the breeding season (6 penguins) or the molting period (12 penguins); and being under treatment for a disease other than aspergillosis (5 penguins). Additionally, only 7 of 54 cases had 2 or more risk factors, indicating that the risk factors were independent (Figure 3).

**Figure 4:**
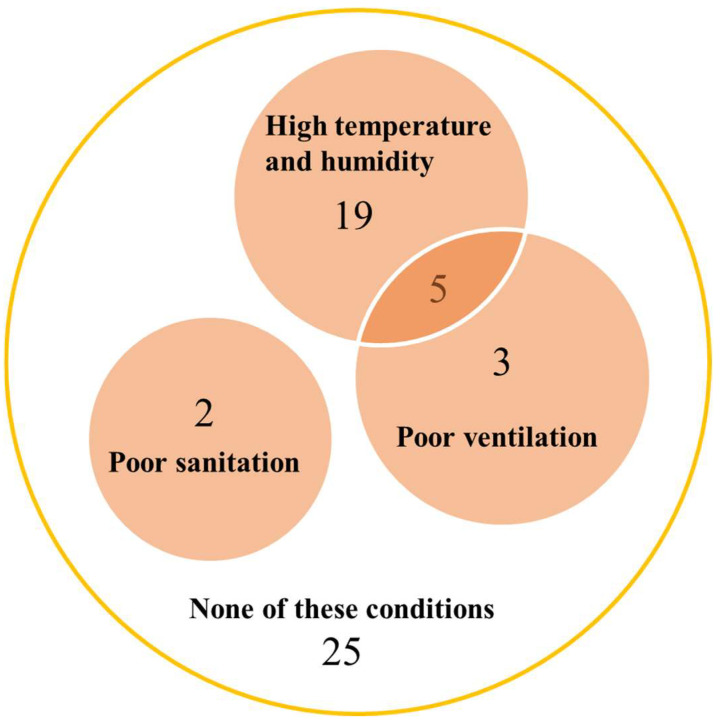
Temporary uncomfortable environmental factors of 54 penguins that died from aspergillosis.

In addition to the individual risk factors described above, we focused on uncomfortable environmental factors surrounding the penguins that presented with aspergillosis. A total of 29 (53.7%) penguins were believed to be reared temporarily in unsuitable environments with poor ventilation (8 penguins), high temperature and humidity (24 penguins), or poor sanitation (2 penguins) (Figure 4). 

**Figure 5:**
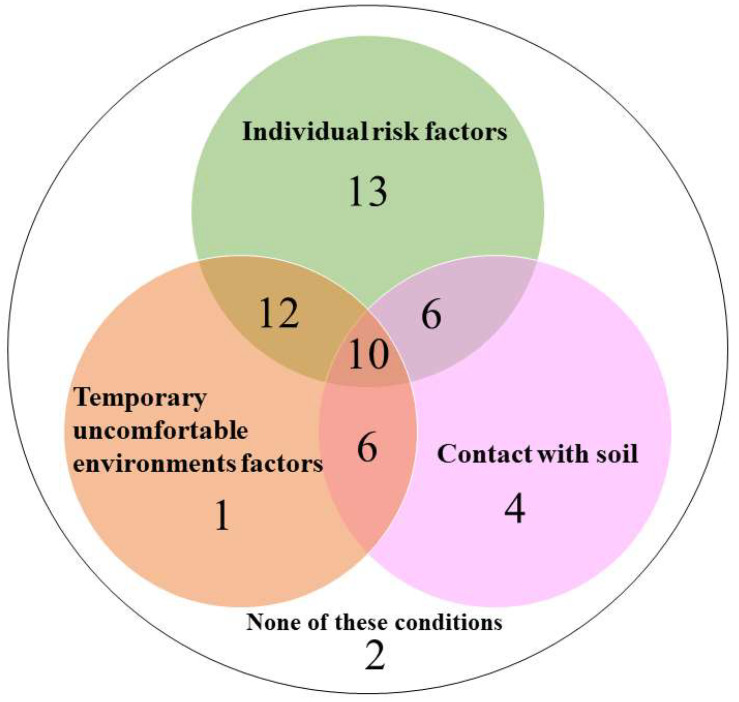
Associations between aspergillosis and individual risk factors, uncomfortable environments factors, and contact with soil in 54 penguins that died from the condition.

Furthermore, 26 penguins (48.1%) had contact with soil. In total, 52 of 54 penguins that died of aspergillosis had at least one of the individual risk factors, uncomfortable environmental factors, and a risk of contact with soil (Figure 5). 

## 4. Discussion

We performed a nationwide survey on the incidence of aspergillosis, rearing conditions, and assessed the measures taken in each rearing facility for penguins in Japan. Furthermore, aspergillosis cases in facilities that responded to the primary questionnaire were investigated with the additional questionnaire particularly focusing on individual and ambient uncomfortable environment factors and contact with soil. Within the previous 5 years, 73 (2.5%) of 2910 penguins had died of aspergillosis. In a 10-year survey from 1960 to 1969 in Japan, 103 penguins were reported to show signs of aspergillosis [32]. Based on the data, the rearing number of penguins each year in Japan was between 194 (1960) and 387 (1968). Compared to the 1960s, approximately seven times or more penguins are currently reared in Japan. The methods of investigation of the earlier research differed from ours; therefore, simple comparisons are difficult to make. However, taking into account the difference in population, it is suggested that the mortality rate is lower nowadays. Although the climate in Japan is hot and humid for penguins, various improvements in zoos and aquariums, such as reference to and adherence to penguin breeding manuals [33] and preventive management [28,29,30], were thought to have reduced the number of deaths. Alternatively, the occurrence of aspergillosis was still found as shown in the survey, suggesting that aspergillosis remains important among facilities rearing penguins in Japan. 

Southern rockhopper penguins, little penguins, and Magellanic penguins had significantly higher mortality rates than other penguins over the last five years. However, aspergillosis cases in southern rockhopper penguins and little penguins were reported in one facility. Moreover, aspergillosis in Magellanic penguins was included in a sporadic outbreak (four of eight deaths from aspergillosis in one facility). Hence, the significantly higher mortality rates were derived from the rearing environment rather than the high species susceptibility. When our analysis excluded data from these facilities, we found no difference in mortality rates according to the penguin species. Prior to this study, we expected that the mortality rates differed between Antarctic/sub-Antarctic species and temperate species because they vary in terms of the recommended range of air and pool water temperatures, as shown in the *Penguin Care Manual* by AZA. These data suggest that the effects of the rearing environment are more significant in the occurrence of aspergillosis than species differences. In accordance with these results, the occurrence of aspergillosis in penguins was strongly related to outdoor enclosures of penguins (*p* < 0.01), moving of penguins outside the rearing enclosures (*p* < 0.01), and contact with soil (*p* < 0.01). These data also indicate the importance of environmental factors in the development of aspergillosis and suggest that the rearing environment is important in reducing the incidence of *Aspergillus* infections.

The occurrence of aspergillosis was significantly higher among the Antarctic/sub-Antarctic penguin species reared outdoors than among those reared indoors, as shown in Table 4. These data indicate that outdoor enclosure is a risk factor for captive Antarctic/sub-Antarctic penguins. However, the analysis has a limitation because it did not confirm whether *Aspergillus* spores in the air or outdoor environment with unsuitable temperature or humidity is a risk factor. We reconsidered if unsuitable temperature and humidity can be a risk factor for the incidence of aspergillosis in an additional survey. The correlation between the occurrence of aspergillosis and contact with soil was not significant for the Antarctic/sub-Antarctic penguin species; however, the number of facilities where these species had access to soil was less. Therefore, further analysis might reveal the correlation. The Antarctic/sub-Antarctic penguins are already recommended to be housed in cooler temperatures than temperate species [3]. 

Aspergillosis was not observed in emperor penguins, chinstrap penguins, or northern rockhopper penguins in this study. A strong possibility for this is the indoor rearing and cold conditions that the penguins were kept in; hence, they had less exposure to *Aspergillus* spores in the air and less heat stress. One Adélie penguin died of aspergillosis, but this was the only case of death due to aspergillosis among 500 penguins at the facility. Using the additional questionnaire, we confirmed that the penguin was only 8 months old. According to a previous report, aspergillosis is common among birds younger than 1 year [7]. Therefore, this penguin was at higher risk of infection compared with adult penguins; it was also reared indoors and had no contact with soil. In such a case, it may be useful to confirm the unsuitable environmental factors. For example, even when housed indoors, air conditioners are associated with a risk of contamination with fungi including *Aspergillus* species. In our previous study [28,29], *A. fumigatus* was detected from air conditioner vents. Moreover, *Aspergillus* is extremely common in the environment, which suggests that clothes and items brought into rearing facilities cause the invasion of *Aspergillus* spores in facilities. The possibility of human-mediated spread was mentioned in a recent study on a mass aspergillosis outbreak in a flightless parrot kākāpō in New Zealand [34]. As a basic preventive measure, cleaning of rearing environments is important.

In contrast to emperor penguins, chinstrap penguins, and northern rockhopper penguins, several king penguins, gentoo penguins, and southern rockhopper penguins died of aspergillosis. In several of the facilities where the aspergillosis occurred, penguins had been kept outdoors and had opportunities to move outside of their housing. In some facilities, penguins also had access to soil. There is no doubt that these penguins were easily exposed to *Aspergillus* spores.

We found that the occurrence of aspergillosis among the temperate penguin species was significantly higher when the penguins were temporarily moved outside of their rearing enclosures and had access to soil. As shown in our previous study, the prevention of access to soil was a useful measure for reducing aspergillosis in an aquarium [28,29]. The results obtained in this study agree with our previous results. Some facilities include soil and plants in outdoor penguin enclosures to resemble their habitats in the wild. Soil is the major habitat of *Aspergillus* [4,14,35]. Therefore, such rearing enclosures might increase the risk of aspergillosis in penguins. Recently, a mass outbreak of aspergillosis in kākāpō (*Strigops habroptilus*) and one case in Okinawa rail (*Hypotaenidia okinawae*) were reported [34,36]. Kākāpō, Okinawa rail, and penguins are flightless birds, and they walk on the soil. In the mass outbreak in kākāpō, a single strain caused aspergillosis, and the association between the ambient environment and aspergillosis was considered an important risk factor [34]. In the case of Okinawa rail, the authors described the environmental source of the causative agent [36]. Although the breeding environments of the Antarctic/sub-Antarctic penguins differed from those of the temperate penguins, contact with soil was considered to increase the risk of developing aspergillosis in both types of species. The trends in temperate penguin species, as shown in Table 4, are similar to those of Antarctic/sub-Antarctic penguin species as discussed above, with out-of-home migration and contact with soil as the major risk factors for aspergillosis development. Even in temperate penguin species, contact with other penguins and soil increased exposure to *Aspergillus* spores, which might be a risk factor for aspergillosis.

In a further analysis of the individual conditions of penguins that died of aspergillosis, 76.0% of them had 1–3 individual risk factors. That is, they were younger than a year, were older than 20 years, or had been transferred from other facilities within the past year. Flach et al. [7] reported that chicks (≤1 year) of gentoo penguins were the most susceptible. Moreover, our data suggest that younger individuals were highly susceptible to aspergillosis. Similar to younger penguins, older penguins might be susceptible to aspergillosis. Keepers and veterinarians also answered that 53.7% of the dead penguins had 1 or 2 uncomfortable environmental factors, such as high temperature, high humidity, poor sanitation, and poor ventilation. These uncomfortable environmental factors were a source of stress for the penguins and a possible risk factor for the development of aspergillosis. Furthermore, 26 of 54 dead penguins had contact with soil, and at least 48.1% of the penguins were at risk of exposure to *Aspergillus* spores. Notably, in 52 of 54 aspergillosis cases, the penguins had one or more individual, unpleasant environmental factors or risk factors of exposure to *Aspergillus* spores.

In this study, only uncomfortable environmental factors were detected in one penguin, and only exposure to *Aspergillus* spores was detected in four penguins. These results suggest that penguins with multiple risk factors are more likely to develop aspergillosis. However, only individual risk factors were detected in 13 penguins. Although multiple risk factors were involved in the development of aspergillosis, other risks were not validated in this study. The current survey had some limitations. Inquiries about ground materials were included in the questionnaire, and soil and stone or sand were counted separately. However, we did not obtain information on the type of soil used for the basement in this survey. As shown in some publications, compost is one of the major sources of *Aspergillus* [37]. Hence, further surveys should examine the type of floor materials to be used in the rearing facility.

Many facilities had implemented various countermeasures to decrease the incidence of aspergillosis among penguins, including prevention, early detection, and preemptive aggressive treatment, of which some are crucial. For example, daily observation, facility disinfection, and body weight measurement are basic preventive measures against not only aspergillosis but also other microbial infectious diseases. However, we could not find a significant improvement even after taking more than five measurements. Therefore, instead of other measures, the risk of acquiring *Aspergillus* spores from the rearing environment must be eliminated. New technologies, such as diagnosis with image analysis [25,27], plasma biomarkers [38], and probiotic-based vaccination [39], might be useful in reducing the incidence of aspergillosis among penguins. However, environmental interventions might be an effective and easy method for decreasing the risk of aspergillosis, which should be considered a priority.

## 5. Conclusions

As demonstrated in this study, aspergillosis was caused by the combination of individual risk factors, such as young and old ages; uncomfortable environments, such as high temperature and humidity; and rearing environments susceptible to exposure to *Aspergillus* spores, such as contact with soil. *Aspergillus* is ubiquitous both in soil and in the air; therefore, it is difficult to eliminate contact with *Aspergillus* outdoors and even indoors. In addition, captive penguins are exposed to hot and humid conditions in Japan, and their immune systems may be weakened as a result of young or old age. However, the death rate is currently much lower, and the preventive measures implemented are effective as basic measures. As further measures, environmental interventions, such as keeping away from soil, might be effective in further reducing the incidence of aspergillosis. Combined measures focusing on individual and environmental risk factors as well as consultation of keepers, veterinarians, and microbiologists, such as mycobiologists, regarding rearing environment can be useful in controlling the incidence of aspergillosis. We believe that our data are informative and will lead to recommendations for further reduction in the number of penguin deaths from aspergillosis.

## Figures and Tables

**Table 1:** The number of facilities and penguins of each species.

	Emperor	King	Adélie	Gentoo	Chinstrap	Northern Rockhopper	Southern Rockhopper	Little	African	Magellanic	Humboldt
Facilities ^†^	2	12	3	14	3	5	7	4	18	12	39
Penguins	23	216	179	359	108	92	79	42	483	242	1087

^†^: The number of facilities was counted for each penguin species, so the total number is more than 64.

**Table 2:** Incidence of aspergillosis in each penguin species over the last 5 years (April 2016–March 2021).

	Emperor	King	Adélie	Gentoo	Chinstrap	Northern Rockhopper	Southern Rockhopper	Little	African	Magellanic	Humboldt
Cases	0	6	1	5	0	0	5	4	10	13	29
Death rate (%)	0	2.8	0.6	1.4	0	0	6.3 *	9.5 *	2.1	5.4 **	2.6
Facilities where aspergillosis occurred	0	4	1	3	0	0	1	1	6	7 ^☨^	19

*: *p* < 0.05, **: *p* < 0.01. ^☨^: High death rate at facility (number of deaths in the facility: 4 (33.3%)).

**Table 3:** Incidence of aspergillosis in all penguin species (119 groups).

		Number of Groups	Number of Groups in Which Aspergillosis Occurred	Number of Groups in Which Aspergillosis Did Not Occur	*p*-Value
Use of outdoor enclosures	Yes	74	35	39	0.0002
	No	45	6	39	
Moving outside of rearing enclosures	Yes	35	19	16	0.003
	No	84	22	62	
Presence of soil	Yes	32	20	12	0.0001
	No	87	21	66	

**Table 4:** Incidence of aspergillosis in Antarctic and sub-Antarctic penguin species (46 groups).

		Number of Groups	Number of Groups in Which Aspergillosis Occurred	Number of Groups in Which Aspergillosis Did Not Occur	*p*-Value
Use of outdoor enclosures	Yes	10	5	5	0.007
	No	36	3	33	
Moving outside of rearing enclosures	Yes	13	4	9	0.143
	No	33	4	29	
Presence of soil	Yes	3	2	1	0.073
	No	43	6	37	

**Table 5:** Incidence of aspergillosis in temperate penguin species (73 groups).

		Number of Groups	Number of Groups in Which Aspergillosis Occurred	Number of Groups in Which Aspergillosis Did Not Occur	*p*-Value
Use of outdoor enclosures	Yes	64	30	34	0.346
	No	9	3	6	
Moving outside of rearing enclosures	Yes	22	15	7	0.009
	No	51	18	33	
Presence of soil	Yes	29	18	11	0.019
	No	44	15	29	

## Data Availability

Restrictions apply to the availability of these data. The data are available from the authors with the permission of the respective zoos and aquariums.

## Supplementary Materials

The following supporting information can be downloaded at: https://www.mdpi.com/article/10.3390/ani13121913/s1, File S1: Primary survey; File S2: Additional survey; File S3: Cases of aspergillosis.
